# Impact of the Controlled Release of a Connexin 43 Peptide on Corneal Wound Closure in an STZ Model of Type I Diabetes

**DOI:** 10.1371/journal.pone.0086570

**Published:** 2014-01-23

**Authors:** Keith Moore, Gautam Ghatnekar, Robert G. Gourdie, Jay D. Potts

**Affiliations:** 1 University of South Carolina School of Medicine, Columbia, South Carolina, United States of America; 2 FirstString Research Inc., Mount Pleasant, South Carolina, United States of America; 3 Medical University of South Carolina, Charleston, South Carolina, United States of America; 4 Virginia Polytechnic and State University Carilion, Roanoke, Virginia, United States of America; Northwestern University, United States of America

## Abstract

The alpha-carboxy terminus 1 (αCT1) peptide is a synthetically produced mimetic modified from the DDLEI C-terminus sequence of connexin 43 (Cx43). Previous research using various wound healing models have found promising therapeutic effects when applying the drug, resulting in increased wound healing rates and reduced scarring. Previous data suggested a rapid metabolism rate in vitro, creating an interest in long term release. Using a streptozotocin (STZ) type I diabetic rat model with a surgically induced corneal injury, we delivered αCT1 both directly, in a pluronic gel solution, and in a sustained system, using polymeric alginate-poly-l-ornithine (A-PLO) microcapsules (MC). Fluorescent staining of wound area over a 5 day period indicated a significant increase in wound closure rates for both αCT1 and αCT1 MC treated groups, withαCT1 MC groups showing the most rapid wound closure overall. Analysis of inflammatory reaction to the treatment groups indicated significantly lower levels of both Interferon Inducible T-Cell Alpha Chemoattractant (ITAC) and Tumor Necrosis Factor Alpha (TNFα) markers using confocal quantification and ELISA assays. Additional analysis examining genes selected from the EMT pathway using RT-PCR and Western blotting suggested αCT1 modification of Transforming Growth Factor Beta 2 (TGFβ2), Keratin 8 (Krt8), Estrogen Receptor 1 (Esr1), and Glucose Transporter 4 (Glut4) over a 14 day period. Combined, this data indicated a possible suppression of the inflammatory response by αCT1, leading to increased wound healing rates.

## Introduction

Type I diabetes, also known as diabetes mellitus, is a common disease characterized by hyperglycemia resulting from a deficiency in insulin production. In this diabetic state, β-cells in the pancreas produce little to no insulin. For the STZ model of type I diabetes, mild to severe diabetic states are induced in a dose dependent manner by targeting the β-cells ability to function normally [1], [2]. Several secondary health problems are commonly associated with diabetes, including risk of stroke, impaired vision, diabetic retinopathy, glaucoma, diabetic ulcers, and decreased wound healing efficiency [3], [4], [5]. Of interest here is impaired wound healing ability. Research has shown decreased leukocyte and macrophage function, difficulties in ECM deposition, and slowed re-epithelialization [6] occurring in diabetic wound healing. The αCT1 peptide is a novel wound healing agent we believe could be effective in treating diabetic wounds.

In vivo, the cytoplasmic tight junction protein zonnula occludin-1 (ZO-1) binds at its PDZ-2 domain with the C-terminus of Cx43 [7], [8]. Previous results studying the Cx43-ZO-1 interaction in the heart found that intact ventricular myocardium exhibited a low level of ZO-1-Cx43 interaction [9]. Wound formation leads to disruption in these interactions, which in turn creates changes in protein-protein interactions, affecting formation of gap junctions. Connexins have also been found to remain in an open state in response to pathology induced stress, including ischemia and hypoxia [10]. To investigate this effect, αCT1, a 25 amino acid sequence comprised of a 16 amino acid antennapedia domain connected to a 9 amino acid (RPRPDDLEI) sequence from the C-terminus of Cx43 was synthesized [11], [12]. Connexins play a key role as mediators of both cell growth and death and function in immune response, hematopoiesis, and development of progenitor cells [13], [14], [15]. Binding of the C-terminus of Cx43 to ZO-1 is believed to affect cellular communication and gap junction remodeling in wound healing [16]. αCT1 interacts with ZO-1 as a competitive inhibitor of this binding [16], [17], increases the rate of wound healing, and reduces scar tissue formation in multiple wound healing models [16], [18], [19]. Recent work by Rhett et al. 2011 indicated that the Cx43-ZO-1 interaction occurs not only at the gap junctions of the plasma membrane, but also the free connexons of the perinexus. A control peptide, with the active C-terminus sequence reversed, was created with the inactive 16 amino acid antennapedia portion unchanged.

Epithelial Mesenchymal transition (EMT) is a biological method of cellular rearrangement and repair of damaged tissue where immobile cells used for structural integrity and boundary formation may be mobilized to an area of need [20]. Once the EMT process is complete the mesenchymal cells differentiate to secondary cell types, such as fibroblasts, or back to epithelial cells in the process called mesenchymal-epithelial transition (MET). Typically the EMT process is tightly regulated by the body, as in embryogenesis [21]. While well understood in most aspects of wound healing, EMT is not as well understood in the eye or in the relationship with wound healing occurring there. However, both Aomatsu et al. 2012 [22] and Kawakita et al. 2012 [23] were able to show the prevalence of EMT in the eye using cornea epithelial cells in relation to TGFβ and Slug signaling. Additional results summarized endothelial mesenchymal transitions occurring in the cornea [24], leading to the belief that EMT occurs in the cornea during healing.

In this study we hypothesized that a controlled dose treatment with αCT1 microencapsulated in A-PLO would produce a significant increase in wound healing when applied to a diabetic corneal injury model. Previous data indicated a rapid metabolism of αCT1 (<2 hrs), leading to an interest in the evaluation of potential therapeutic advantages possible with controlled release. Additionally, previous studies have not completely shown the method of peptide action. Here we examine genes from the EMT pathway, selected for their relationship to potential points of peptide influence, such as proliferation, motility, inflammatory regulation, and morphogenesis. In vitro experiments probing control and αCT1 treated bone marrow stromal cell monolayers also indicated potential genes of interest; Krt8, Krt19, and TGFβ2. We also evaluated the roles of EMT associated genes related to insulin sensitivity by examining GLUT4 and Esr1.

## Materials and Methods

### Induction of Type I Diabetes, Corneal Surgery, and Wound Treatment

Induction of type I diabetes was performed by single dose I.P injections of STZ (Sigma Aldrich) at 65 mg/kg in Sodium Citrate buffer pH 4.5 (Sigma Aldrich), given to male Sprague-Dawley rats (Harlan Laboratories) weighing 240–260 grams, in a manner based on previously published protocols [4], [25], [26]. After injection, a one week period was allowed for complete diabetic illness to take effect. At the end of the one week period rats were fasted overnight (18 hrs) then tested for blood glucose levels from the tail vein using a ReliOn Confirm blood glucose meter and ReliOn ultra-thin lancets. Rats with blood glucose levels exceeding 225 mg/dL were considered diabetic and used in this study. Food and water intake, as well as animal health was monitored throughout the study. Animals expressing discomfort or poor health were removed.

All surgeries were performed in strict compliance with the Guide for the Care and Use of Laboratory Animals by the National Academy of Sciences and University of South Carolina Animal Resources Facility guidelines. The protocol was approved by the Institutional Animal Care and Use Committee at the University of South Carolina School of Medicine (protocol #2037-100393-093011). Rats were placed under anesthesia using 60 mg/kg Ketamine (Putney Inc), 7.5 mg/kg Xylazine (Lloyd Laboratories), and 1 mg/kg Acepromazine (Phoenix Pharmaceuticals). Two drops of Alcaine (Alcon Canada, Mississauga, Canada) topical anesthetic were applied to each eye, then removed with a sterile ophthalmic sponge (Merocel, Beaver-Visitec International). A 5 mm trephine was placed on the cornea with two drops of 20% isopropyl alcohol added to the center, touching the cornea surface for exactly 30 seconds. Care was taken to create wounds affecting the central cornea and avoiding the limbal region, which could affect healing. Exposure to the alcohol solution loosened a 5 mm area of corneal epithelium, which was then removed by gentle scraping, leaving the exposed stromal layer. The cornea was rinsed with 1% saline to remove any remaining loosened epithelium.

Post surgery, rats were placed into one of four time point groups spanning a two week period; 1 day, 3 days, 5 days, or 14 days. These four time points were repeated for four separate treatments, with a total of 80 rats used in the study (20 per treatment/5 per time point). At each time point a total of 10 eyes were used with identical treatment in both eyes of each rat to eliminate eye to eye cross contamination. Bilateral surgeries were deemed appropriate in this study based on the lack of a permanent or long term debilitating injury being induced on the eye. Furthermore, animals were carefully monitored throughout the timespan of the project for evidence of distress or behavior which would indicate their need for removal from the dataset (i.e. inability to eat, drink, or groom properly). Animals were ethically sacrificed by a combination of carbon dioxide exposure and cervical dislocation, followed immediately by sterile whole eye removal. Each treatment group contained two controls (pluronic gel alone; control peptide) and two treatments with αCT1 peptide (direct application in pluronic gel; αCT1 A-PLO microcapsules). To ensure sustained delivery of peptide into the corneal wound, a pluronic gel carrier (Pluronic F-127, Sigma Aldrich) was used at a concentration of 25% w/v. Control group rats received treatments of either 10 µl 25% pluronic gel or 10 µl of 150 µM control peptide/25% pluronic gel. Application of αCT1 was again delivered at either 10 µl 150 µM αCT1/25% pluronic gel per eye or 150 µM αCT1 Microcapsules/10 µl 25% pluronic gel per eye with A-PLO microcapsules. Each treatment was applied immediately after surgery (0 hrs), at 24 hrs, and at 72 hrs. All rats were ethically sacrificed at the designated endpoints.

### Synthesis of αCT1 Loaded A-PLO Microcapsules

Microcapsules were synthesized according to a previously published protocol [27]. Sterile sodium alginate (Sigma-Aldrich catalog#A0682, high maluronic acid content, low viscosity) poly-l-ornithine (Alfa Aesar L-ornithine hydrochloride 99%) microcapsules were synthesized by the electrospray method. All microcapsules were synthesized with a 2% alginate/0.5% PLO concentration and gelled in 0.15 M calcium chloride (CaCl_2_) (Sigma-Aldrich) solution for 12 minutes. The synthesis parameters were constant with a needle to working bath distance of 7 mm, a voltage of 6.0 kV, and a flow rate of 60 mm/hr. Microcapsule solutions were buffered to pH 4.3 using 0.1 M HEPES and 0.1 M hydrochloric acid (HCL) to control pore size and slow release of αCT1. A-PLO microcapsules were loaded with αCT1 at an initial concentration of 200 µM. Post synthesis the peptide release profile was determined by rinsing three times in deionized water, incubation at 37°C in deionized water, and measurement of peptide concentration in solution using a microBCA Assay kit (Thermo Scientific) over 48 hrs. Using these parameters a microcapsule releasing ∼150 µM αCT1 (established therapeutic range) over 48+hrs was synthesized.

### Wound Measurements and Analysis

Analysis of the change in wound closure rate over time was performed by adding 2 µl drops of Fluress (fluorescein sodium and benoxinate hydrochloride, Akorn Incorporated) GFP labeled fluorescent dye to the wounded cornea with coverage of the eye surface. Excess dye was removed, leaving a thin coat attached to the wounded cornea. Images were taken of the wounds at 0 hrs (immediately post surgery), 1 day, 3 days, 5 days, and 14 days, using a Zeiss Lumar V12 fluorescence microscope with a 20X objective and 1.5X lens magnification. All images were taken prior to application of treatments. The area of each wound site was measured using Axiovision Release 4.8.2 software.

### Confocal Quantification of Inflammation Using ITAC and TNFα

Cornea samples from all four treatments were fixed overnight at 4°C in 2% paraformaldehyde. Fixed samples were then stained for immunohistochemistry using antibodies for ITAC and TNFα according to a previously published protocol [28] (See Table 1). Staining was carried out 1 hr at RT for both ITAC and TNFα on samples from all four treatments at time points of 1 day, 3 days, and 5 days. Nuclear staining with DAPI diluted 1∶2000 was also performed for 30 minutes. Alexa Fluor 488 (TNFα) or 546 (ITAC) (Molecular Probes, Invitrogen) secondary antibodies were then added at 1∶100 dilutions in blocking buffer 1 hr. Images were obtained with a Zeiss LSM 510 Meta CSLM microscope of two separate regions within the wound margin where inflammation fluorescence was visualized for both ITAC and TNFα. Pluronic gel treated samples from the 1 day time point were used to initially set the upper threshold settings for each inflammatory marker. The reuse setting of the microscope was then applied to all subsequent images, which allowed identical microscope conditions at each treatment and time point. Additionally, all samples were stained and imaged under identical conditions and at the same time to eliminate variation in processing and photo bleaching over time. Negative controls were created using samples stained with only secondary antibodies.

**Table 1:** Summary of antibodies used for confocal inflammatory quantification.

Primary Antibodies	Dilutions	Manufacturer	Purpose of Interest
DAPI (4′,6-diamidino-2-phenyl-indole)	1∶2000	Invitrogen	Nuclei marker
TNF-α	1∶100	Phar Mingen	Inflammatory Cytokine
Anti-mI-TAC (CXCL11)	1∶100	R&D Systems	Chemokine/activated T cells

Images were next analyzed using the ImageJ 1.45 s software package (National Institutes of Health, Bethesda, Md). Measurements were set to analyze mean intensity with limits to threshold and the threshold adjusted to create a binary image of the stained areas of inflammation. Binary images were then processed using the smooth function to solidify the edges. Each measurement was performed in triplicate and recorded. The measurements were converted to percent of total coverage using the formula 100-((x-255)*100), with x representing the measured value and 255 the maximum threshold limit

### ELISA Using the Inflammatory Marker TNFα

To validate the confocal inflammatory quantification data, ELISA (enzyme linked immunosorbant assay) was performed using the TNFα cytokine marker. Wounded cornea samples were dissected from all four treatment groups at 1, 3, and 5 days. Samples were homogenized and placed in a solution of T-PER tissue protein extraction reagent (Thermo Scientific) and Halt protease inhibitor (Thermo Scientific). The solutions were centrifuged at 10,000 g for 5 minutes. The resulting protein supernatant was analyzed for total concentration using a Coomassie assay (Thermo Scientific) according to manufacturer's instructions on a BioTek Synergy 2 spectrophotometer. ELISA was then conducted in triplicate on protein samples using a rat TNFα ReadySET-Go! (eBioscience) kit according to manufacturer's instructions.

### RT-PCR of EMT Genes

RT-PCR was performed in the same manner as described in a previous protocol [28]. Corneas were sectioned at 1, 3, and 14 day time points for each of the pluronic gel, control peptide, αCT1, and αCT1 A-PLO microcapsule treatments. Entire corneas were cut into sections, with individual ¼ sections placed in 1 mL of Trizol Plus RNA purification reagent (Invitrogen), and processed according to the manufacturer's protocol. Extracted RNA concentration was measured using an Agilent 2100 bioanalyzer and Agilent RNA 6000 nano kit (Agilent Technologies, Inc). Two samples of RNA per treatment type were selected for each of the three (1, 3, 14 days) time points, ensuring no RNA samples were selected from the same rat source in the same treatment group. These samples were then converted to cDNA at 250 nM using a BioRad iScript cDNA kit (Bio-Rad Laboratories) according to manufacturer's instructions. Gene specific primers for Keratin 19, Keratin 8, and TGFβ2 of the EMT pathway and the insulin sensitive genes Esr1 and GLUT4, related to the EMT pathway were designed using BLAST web based software and synthesized by IDT technologies (Table 2). The gene attachment region binding protein (ARBP) was used as a calibrator reference. Primers were tested for efficiency using RNA from day 3 pluronic gel treated diabetic rats and optimized by PCR gel analysis (data not shown).

**Table 2:** Summary of primers used in RT-PCR analysis.

Gene	Forward Primer	Reverse Primer	Product Length (bp)
ARBP	5′-CGACCTGGAAGTCCAACTAC-3′	5′-ATCTGCTGCATCTGCTTG-3′	109
Krt19	5′-AGCATGAAAGCTGCCCTGGAA-3′	5′-ATACTGCTGATCACACCCTGGA-3′	92
GLUT4	5′-CTCTCAGGCATCAATGCTGTT-3′	5′-GAGACCAACGTGAAGACGGTA-3′	122
Esr1	5′-AGTGAAGCCTCAATGATGGG-3′	5′-CAAAGATCTCCACCATGCCT-3′	281
Krt8	5′-CTTCTCCCTAGTCACCCACT-3′	5′-TTCCATGTTCGGTCTGCTTC-3′	110
TGFβ2	5′-GCCAATGTAGTAGAGGATGGCTC-3′	5′-AAACTAACCACTTTCTTGCGTG-3′	108

Real-time PCR was run with each gene according to a previously published protocol [28] using a BioRad CFX Connect single color real time PCR detection system (Bio-Rad Laboratories) with SsoAdvanced SYBR Green supermix, 1 µl cDNA, and 3 pmol/rxn primers.

### Western Blotting of EMT Markers

Wounded cornea samples were dissected from all four treatment groups at 1, 3, and 14 days. Samples were homogenized and placed in a solution of T-PER tissue protein extraction reagent (Thermo Scientific) and Halt protease inhibitor (Thermo Scientific). The solutions were centrifuged at 10,000 g for 5 minutes. Resulting protein supernatant was analyzed for total concentration using a Coomassie assay (Thermo Scientific) according to manufacturer's instructions on a BioTek Synergy 2 spectrophotometer.

Solutions of 20 ug/lane of protein mixed 1∶1 with Laemmeli buffer/β-mercaptoethanol were denatured at 70°C, then loaded onto BioRad Criterion 4–15% TGX 12+2 gels, with BioRad Precision Plus loaded as a standard. SDS-Page was performed at 200 V in Tris/Glycine/SDS buffer (Biorad). Gels were next wet transferred by western blotting onto supported nitrocellulose membranes (BioRad) at 100 V in transfer buffer (Tris/Glycine buffer (BioRad) with methanol pH 8.0). Blotted membranes were blocked for 1 hr in a solution of 5% non-fat dry milk (BioRad) and PBS-Tween (Sigma), rinsed three times in PBS-Tween, and stained overnight at 4°C with primary antibodies (Table 3). Antibodies were selected to match genes tested for RT-PCR; Krt19, Krt8, TGFβ2, Esr1, and GLUT4. The GAPDH antibody was used as a reference control. Blots were then rinsed three times in PBS-Tween and stained 1 hr with secondary HRP labeled antibodies (Invitrogen). Blots detected by Hyglo chemiluminescent HRP detection reagent (Denville Scientific Inc.) and images captured using a BioRad ChemiDoc XRS+ system with QuantityOne software. Bands were standardized against GAPDH and measured for density with equal volume boxes applied to each blot to standardize total area. Each time point was performed in duplicate for every antibody. To compare the results from blot to blot, measurements were converted to values representing fold change analyzed against pluronic treated samples for 1 day, 3 day, and 14 day times.

**Table 3:** Summary of antibodies used for western blotting.

Primary Antibodies	Dilutions	Manufacturer
Krt19	1∶500	Novus Biologicals
Krt8	1∶10000	Novus Biologicals
TGFβ2	1∶500	Santa Cruz Biotechnology
Esr1	1∶500	Santa Cruz Biotechnology
GLUT4	1∶500	Santa Cruz Biotechnology
GAPDH	1∶500	Santa Cruz Biotechnology

### Statistical Analysis

All graphing was performed using GraphPad Prism 5 software. Data was normalized prior to statistical analysis. The percent decrease in wound area was calculated for each trial and treatment by comparing differences in wound area between initial and final measurements taken at each time point. Analysis for significance was performed analyzing the percent difference vs pluronic treated control groups and Kaplan-Meier survival analysis. Statistical analysis was conducted using two way ANOVA and the Mantel-Cox test. Confocal values were also analyzed by two way ANOVA, comparing control peptide, αCT1, and αCT1 MC groups against pluronic treated samples. Elisa protein concentrations were measured and statistical analysis performed using two way ANOVA, comparing against pluronic treated samples as well. RT-PCR data was analyzed using the Relative Expression Software Tool (REST XL), with pluronic gel treated samples serving as the baseline control. Statistical analysis was performed on the RT-PCR data using both the REST XL Pair Wise Fixed Reallocation Randomization Test and GraphPad Prism data analysis software. Western Blotting results were analyzed using two way ANOVA. In all statistical analysis p<0.05 was set as significant.

## Results

### Analysis of Diabetic Corneal Wound Closure

**Figure 1:**
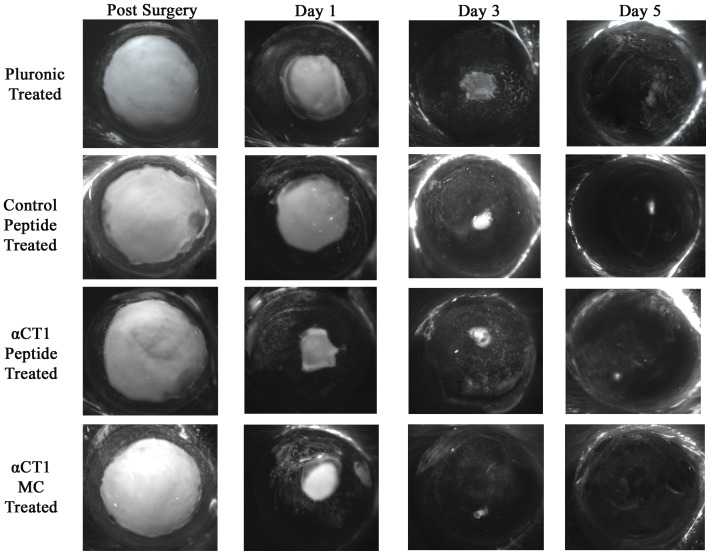
Visual progression of corneal wound closure using Fluress ophthalmic dye staining of corneal defects immediately after surgery and repeated at 1 day, 3 days, and 5 days. Treatments from top to bottom on each eye: 25% pluronic gel, 150 µM control peptide, 150 µM αCT1, and 150 µM αCT1 MC.

**Figure 2:**
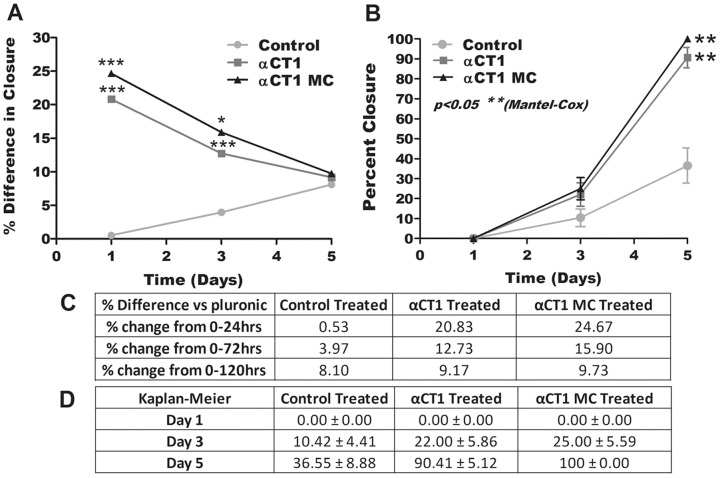
Diabetic corneal wound closure statistical analysis of changes in wound area after injury. (**A**) Summary of the average percent differences in wound closure vs pluronic controls over the first 5 days. (**B**) Kaplan-Meier survival analysis modified for percent closure over a 5 day period. Summaries of individual values ± SEM for the graph in **A** are found in **2C** and values for **B** are found in **2D**.

Examination of wound closure rate was conducted through fluorescent staining with an ophthalmic dye and subsequent measurements of the changing area of the corneal wounds. A total of 25 eyes with measurements spanning the time points at 0 hrs, 1 day, 3 days, and 5 days were selected from each treatment group to determine the average change in wound area over time. Additional measurements were also performed at 14 days, but were excluded after near 100% closure was seen in all eyes by day 5. Figure 1 provides a visual summary of the wound closure rate while table 4 lists the average values for each treatment over the 5 day (120 hrs) period as percent change in wound area (± SEM) relative to pluronic controls. From this data we were able to conclude αCT1 treatment led to a faster wound closure rate than control treatments, while αCT1 MC treatment showed even greater wound closure speed. Statistical analysis of this data was conducted through two way ANOVA analysis of the differences in average wound closure vs pluronic treatment (p<0.05). Figure 2A represents this data graphically, while Figure 2C contains the specific numerical results. Significant differences in wound closure were found in both αCT1 and αCT1 MC treatments at 1 day (20.83% and 24.67% respectively) and 3 days (12.73% and 15.90% respectively). In comparison no time points were found significant for control peptide treatments. To further analyze the significance of the differences in both αCT1 and αCT1 MC treated wound closure we conducted a Kaplan-Meier survival analysis replacing percent closure with survival. The results of this study are presented in Figure 2B and numerically in Figure 2D. The curve pattern was conserved between the Kaplan-Meier and the percent difference data. From Figure 2D we saw, by the 5 day period, a complete closure of the wounds in all eyes for the αCT1 MC treated rats. The αCT1 treated wounds were at 90.41% closure, while control peptide treated were 36.55% closed. Mantel-Cox analysis comparing the curves in figure 2B showed both αCT1 and αCT1 MC treated curves to be significantly different compared to control peptide treated.

**Table 4:** Summary of the percent change in wound closure rate measured using Fluress ophthalmic dye over 5 days.

	Pluronic Treated	Control Peptide Treated	αCT1 Treated	αCT1 MC Treated
% change 0–24 hrs	44.69±21.34	45.22±22.80	65.52±20.15***	69.36±14.02***
% change 24–72 hrs	73.71±26.60	80.97±18.79	87.12±18.70	97.67±4.27
% change 0–72 hrs	83.04±21.43	87.01±16.09	95.77±5.44*	98.94±2.10***
% change 0–120 hrs	90.27±13.50	98.37±1.42	99.44±1.84	100±0.00

Total wound area measured using Axiovision software with analysis of the total change in wound area in the range of time indicated. Each data point represents 25 randomly selected eyes from each treatment ± SEM. Grubb's test for outliers used to remove non-significant data points (p>0.05).

### Investigation of Inflammation Using Confocal Quantification and ELISA

**Figure 3:**
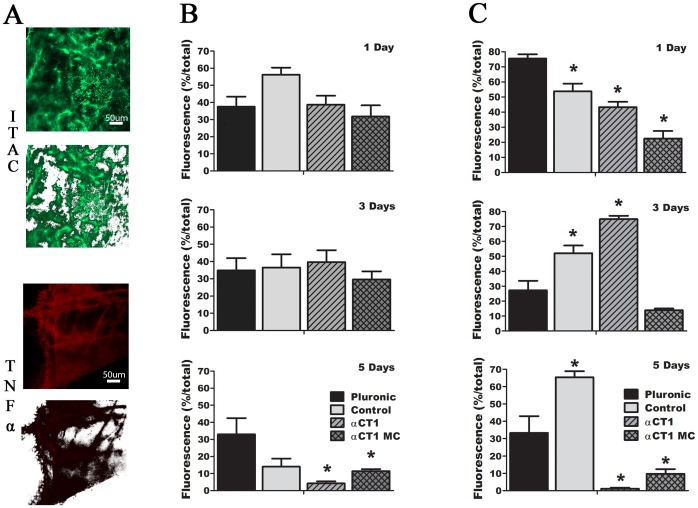
Inflammatory quantification by confocal microscopy. Images and measurements were taken using identical treatments and microscope parameters of regions immediately adjacent to the wound margins with analysis using ImageJ software. (A) Examples of inflammation seen at 3 days along the wound margin for both ITAC (upper) and TNFα (lower) are shown both before (top) and after (bottom) ImageJ thresholding. The results of quantification of 12 images per time point and treatment at days (from top to bottom) 1, 3, and 5 using antibodies for (**B**) ITAC and (**C**) TNFα. Data represents the fluorescence measured as a percent of the total image area, using the presence of fluorescence as an indicator of inflammation.

We next set out to examine the effects of each treatment on inflammation over the first five days of wound closure. Two markers of inflammation, ITAC (CXCL11) and TNFα, were selected to stain representative cornea samples at 1, 3, and 5 days. Using ImageJ software, mean intensity values were measured and converted to percent coverage of the entire image. A total of 12 measurements per inflammation marker were analyzed with average values shown in Figure 3.

The results of ITAC examination at day 1 indicated ∼30–40% total coverage for all treatments except control peptide (56.23±4.07%). By day 3 this trend continued with ∼30–40% coverage seen in all treatment groups. By 5 days, as complete wound closure was approached, only pluronic treated corneas still showed values in the 30–40% range (32.96±9.49%), with control peptide dropping to 14.05±4.72%. Both αCT1 and αCT1 MC treated corneas showed significant reduction (p<0.05) compared to pluronic treated with values of 4.23±1.22% and 11.39±1.16% respectively. Data from the confocal TNFα quantification showed values that were different overall, but shared a similar trend over the same three time points. At 1 day both αCT1 (43.29±3.53%) and αCT1 MC (22.49±5.02%) treated corneas showed values lower than those seen in both controls, with both significantly different compared to pluronic treated. The pattern of expression with TNFα was the same as ITAC at 3 days, with a spike in αCT1 (74.91±2.16%) producing the highest levels. This was followed by pluronic treated (27.24±6.40%), control treated (52.12±5.11%), and finally αCT1 MC treated (13.89±1.12%) at the lowest expression. By day 5, pluronic and control peptide treated corneas continued to show the highest values; similar to patterns seen in the day 5 ITAC results. Additionally, αCT1 and αCT1 MC treated samples again showed significantly lower expression vs pluronic at 5 days, matching the results from 5 day ITAC evaluation as well.

**Figure 4:**
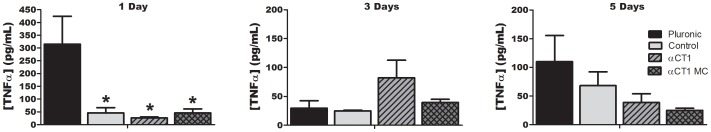
Elisa analysis of TNFα concentration in 1, 3, and 5 day corneal protein samples. Values represent protein extracted from representative wounded cornea samples dissected to contain the central wounded cornea regions only. Statistical analysis by two way ANOVA (p<0.05) comparing against pluronic treatments at each time point.

To corroborate the confocal quantification data, ELISA analysis using antibodies for TNFα was performed, with results shown in Figure 4 and Table 5. Day 1 ELISA results followed the same pattern as those seen in confocal quantification, with pluronic treated samples having the highest concentration. Both αCT1 (26.08±3.50 pg/mL) and αCT1 MC (46.40±15.23 pg/mL) treated corneas showed values lower than those seen in both controls, with both being significantly different compared to pluronic treated. By day 3, αCT1 treated samples again showed a spike while the other three treatments results varied in comparison to confocal quantification analysis. Day 5 analysis saw no significant changes in the results of αCT1 and αCT1 MC treated samples in comparison to the controls, but overall these two active treatments were found to once again be lower at this time than both control groups.

**Table 5:** Summary of TNFα Elisa results for 1, 3, and 5 day samples.

	Pluronic Treated	Control Peptide Treated	αCT1 Treated	αCT1 MC Treated
**Day 1**	315.15±109.70	45.51±20.90*	26.08±3.50*	46.40±15.23*
**Day 3**	29.80±12.43	24.94±0.94	82.21±30.42	39.58±5.39
**Day 5**	110.01±45.47	68.16±23.89	38.61±15.33	25.14±3.47

Results are the average concentration of TNFα in pg/mL ± SEM.

### Examination of the EMT Pathway in Diabetic Corneal Wound Healing by RT-PCR and Western Blotting

**Figure 5:**
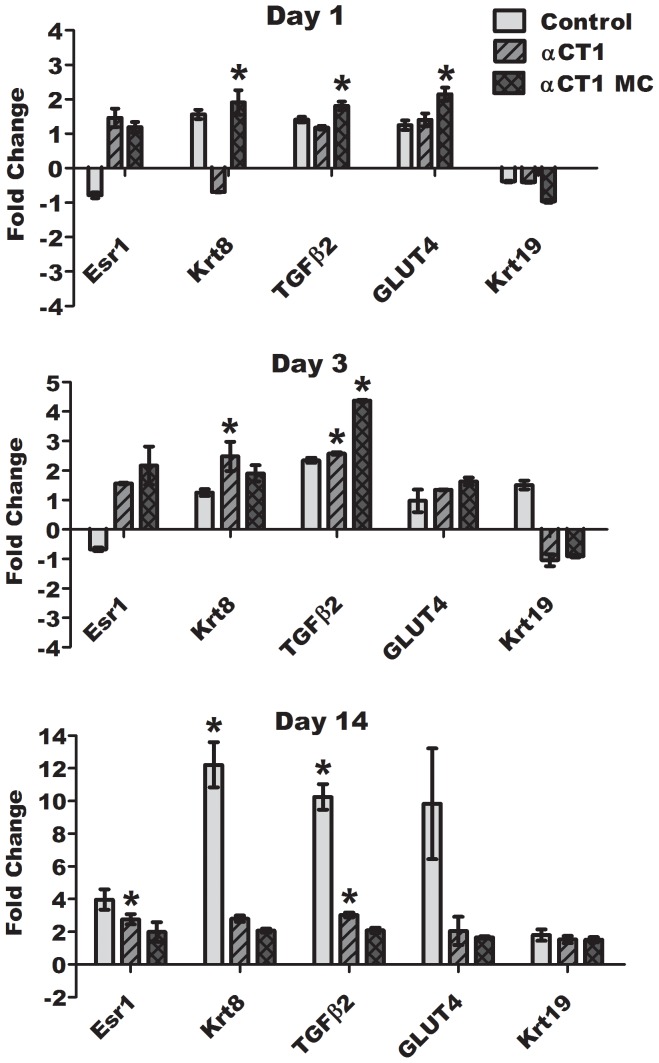
RT-PCR examination of EMT and insulin sensitive genes in 1, 3, and 14 day wounded diabetic corneas. ARBP used as a reference gene with pluronic treated samples serving as baseline controls. RNA extracted from representative corneas dissected to contain only the central wounded section of the cornea. Statistical analysis by Pair Wise Fixed Reallocation Randomization Test (p<0.05).

Examination of both wound closure and inflammation data indicated a noticeable effect on corneal wound healing with both αCT1 treatments. RT-PCR and western blotting was performed on a set of genes selected from the EMT pathway and related genes known to be insulin sensitive as a means of investigating possible reasons for the therapeutic results. Figure 5 summarizes the results of the RT-PCR analysis of the five genes of interest. A consistent up regulation of Esr1, Krt8, TGFβ2, and GLUT4 at days 1, 3 and 14 is seen, while Krt19 was down regulated at days 1 and 3. Significance, determined by a pair wise fixed random reallocation test, was found for only αCT1 MC treatments at day 1 in Krt8, TGFβ2, and GLUT4. At day 3 significant up regulation was found in both αCT1 and αCT1 MC treated samples with TGFβ2 and significance in Krt8 for αCT1 only. By day 14 all genes were up regulated, with TGFβ2 and Esr1 significant in αCT1 treated samples. All genes that were control peptide treated showed strong expression relative to both αCT1 and αCT1 MC groups at day 14.

**Figure 6:**
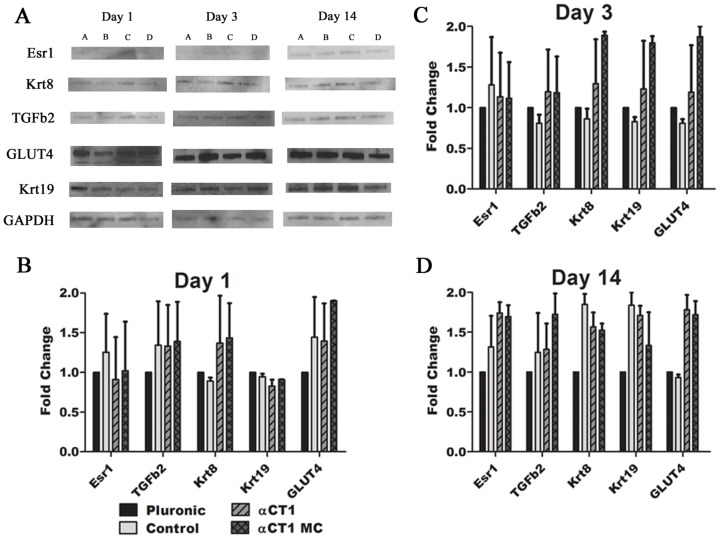
Western blot densitometric analysis of EMT and insulin sensitive genes in 1, 3, and 14 day diabetic corneas. (**A**) Each blot represents from left to right: A:Pluronic treated, B:Control Peptide treated, C:αCT1 treated, D:αCT1 MC treated at the indicated time points for each marker. Summaries of densitometric analysis of the blots is shown for the (**B**) 1 day (**C**) 3 day and (**D**) 14 day samples. GAPDH used to normalize all results. Protein was extracted from representative corneas dissected to contain only the central wounded section of the cornea.

Figure 6 represents the results of densitometry analysis of western blots from 1, 3, and 14 day samples for the same genes evaluated by RT-PCR. All results are presented as fold change differences vs pluronic treated samples. GAPDH was used as the internal control antibody for normalization. In all time points and treatments no values were found to be statistically significant fold changes in protein expression compared to pluronic controls, however similar patterns of regulation matched the results found in RT-PCR quantification. Data from all three time points indicated similar up regulation of TGFβ2, Krt8, and GLUT4 for αCT1 and αCT1 MC samples, as seen in the RT-PCR data. TGFβ2 was again up regulated through all three time points with values between ∼1.2–1.4 fold different than pluronic treated, except at day 3 in control treated samples. Krt8 was inconsistently up regulated with biphasic results in the RT-PCR data, but significant up regulation was shown in αCT1 MC treated samples at 1 day and αCT1 treated samples at 3 days. Densitometry measurements at 1 and 3 days showed Krt8 up regulated in αCT1 (1 day– 1.37, 3 days–1.30 fold up regulated) and αCT1 MC treated (1 day– 1.43, 3 days– 1.89 fold up regulated) samples compared to both pluronic and control peptide treated. Similar to the RT-PCR results, Esr1 showed consistent up regulation in the western data, with particularly higher results in day 14 samples treated with αCT1 (1.74 fold) and αCT1 MC (1.70 fold) treatments. Krt19 western analysis indicated down regulation at 1 day for all three treatments, similar to the RT-PCR results. This deviated at days 3 and 14 with up regulation in the western analysis while the RT-PCR data showed down regulation at 3 days and up regulation at 14 days for all treatments. GLUT4 analysis remained the same in both western and RT-PCR analysis pattern, where all treatments were up regulated throughout the three time points.

## Discussion

Corneal wound healing was found to approach complete closure in all four treatment groups by the 5 day time point, with pluronic gel treated samples averaging ∼90% closure, αCT1 ∼99%, and αCT1 MC 100%. Statistical analysis using two way ANOVA to compare the percent differences in closure at each time point showed significant differences at days 1 and 3 for both αCT1 (20.83% and 13.41% faster than pluronic treated) and αCT1 MC (24.67% and 23.96% faster than pluronic treated) treatment groups. Additional statistical analysis using the Kaplan-Meier method with analysis by the Mantel-Cox test, comparing survival distributions of two samples, also indicated significant differences in the wound healing curves of each of the two αCT1 treatments. The Kaplan-Meier method is typically used to estimate survival over time in cases such as cancer treatments in large groups of patients, while also accounting for difficulties and changes from patient to patient. This method is used to generate survival curves based on designations of alive/dead status in each patient, followed by analysis of statistical differences in their patterns. Here this method was modified to replace alive/dead with open/closed in relation to corneal wound state. Doing so, we were able to analyze the probability of these curves occurring in larger studies. Overall these results indicated application of the αCT1 peptide significantly enhanced wound closure in a corneal injury model. Additional therapeutic effects in the form of increased wound healing rate (3.84% at 24 hrs vs αCT1 alone) were shown to occur with the addition of controlled release A-PLO MC containing αCT1. These results match the wound healing patterns previously seen in non-diabetic corneal wound closure evaluated in our previous studies. Comparing the differences in closure between αCT1 and αCT1 MC treatments at each of the three measured points, 1, 3, and 5 days, there was a consistently faster rate of closure in microencapsulated samples (3.84%, 3.17%, and 0.56% respectively). This led us to conclude A-PLO microencapsulated αCT1 provided a definite advantage in therapeutic treatment over direct αCT1 treatment.

Investigation of the mechanisms of αCT1 action to create enhanced wound healing speed was examined by looking at inflammatory levels over time and gene analysis from the EMT pathway. The results of the Western blotting and RT-PCR data indicated a relationship among genes that were up regulated significantly with αCT1 treatments in comparison to control treatments. Each of these genes are known to play either a direct or indirect role in inflammatory regulation. From the RT-PCR and western analysis, Krt8 and TGFβ2 were consistently up regulated for both αCT1 and αCT1 MC treatments at the three time points. Interestingly, TGFβ2 was the only gene up regulated throughout all points. This cytokine directly limits the inflammatory response, promotes accumulation and proliferation of fibroblasts, and controls deposition of ECM for proper tissue repair [29]. Specifically, Huh et al. 2009 [30] found TGFβ2 was highly expressed during chick corneal wound repair. Their data suggest TGFβ2 activates keratocytes to transform into fibroblasts, a known action of wound healing in the cornea. These fibroblasts are then reverted back to keratocytes as the wound closes. Keratins are tissue specific proteins typically associated with the cytoskeleton of epithelial cells. The estrogen receptor pathway is directly related to insulin sensitivity, type I diabetes, and the EMT process. Krt19 is thought to be down regulated in EMT [31] and related to the estrogen receptor pathway along with Esr1. Both Krt8 and Krt19 have been shown to be present in the limbal epithelial cells of the cornea and are normal markers in healthy cells [32], [33], [34], [35]. The Esr1 gene, or ERα, is important in protection against and reduction of inflammation, as well as glucose tolerance [36], [37]. Glucose transporter 4, or GLUT4, in vivo levels are altered by the inflammatory activation of stress kinases [38]. This action is impaired in diabetes and obesity.

Both αCT1 treatment groups saw a significant decrease in inflammation by day 5 as the wounds approached complete closure. Specifically looking at the day 1 TNFα confocal results, we saw identical highest to lowest order between inflammation level and day 1 wound closure. During this rapid wound closure period, pluronic treated samples were shown to exhibit the most inflammation and the least change in wound closure. This was followed in order by control peptide treated, αCT1 treated, and αCT1 MC treated, observing a direct coupling of inflammation levels to wound healing rate. A deviation in this exact pattern was found at day 3, with a spike in αCT1 levels for ITAC and TNFα. Overall when comparing the results at all three points for ITAC, TNFα and Elisa TNFα there was a pattern of lowered inflammation with αCT1 and microencapsulated αCT1 treatments compared to control groups, which corresponded to the rate at which corneal wound closure occurs.

We saw a mode of action where αCT1 stimulates multiple proteins responsible for inhibition of inflammation. Combined with our inflammatory data investigating markers for ITAC and TNFα, αCT1 is thought to increase wound healing rates by creating a complex reduction in inflammation that is significantly different than control treatments. This reduced inflammatory response may allow a more rapid induction of epithelial migration and proliferation. Additional investigation of this reaction to αCT1 is required to make definitive statements, with a complex response to the peptide likely.

### Conclusions

Treatment of diabetic corneal wound injuries with αCT1 peptide increased wound closure speed significantly compared to control groups. The addition of a polymeric αCT1 A-PLO microcapsule system further increased the wound healing speed over αCT1 alone. A possible mechanism of αCT1 action was suggested, based on inflammatory and RT-PCR investigation of key EMT genes, indicating the activation of anti-inflammatory cytokines and proteins. Suppression of inflammation may lead to a more rapid proliferation and migration response to wound closure in the cornea.
